# A Skin-Inspired Stretchable, Self-Healing and Electro-Conductive Hydrogel with a Synergistic Triple Network for Wearable Strain Sensors Applied in Human-Motion Detection

**DOI:** 10.3390/nano9121737

**Published:** 2019-12-06

**Authors:** Yuanyuan Chen, Kaiyue Lu, Yuhan Song, Jingquan Han, Yiying Yue, Subir Kumar Biswas, Qinglin Wu, Huining Xiao

**Affiliations:** 1 College of Materials Science and Engineering, Joint International Research Lab of Lignocellulosic Functional Materials, Nanjing Forestry University, Nanjing 210037, China; yuanyuanchen1029@163.com (Y.C.); forlovever@outlook.com (K.L.); yuhansong1986@outook.com (Y.S.); 2 College of Biology and Environment, Nanjing Forestry University, Nanjing 210037, China; yue@njfu.edu.cn; 3 Laboratory of Active Bio-based Materials Research Institute for Sustainable Humanosphere, Kyoto University, Uji, Kyoto 611-0011, Japan; subir.biswas.88a@st.kyoto-u.ac.jp; 4 School of Renewable Natural Resources, Louisiana State University, Baton Rouge, LA 70803, USA; wuqing@lsu.edu; 5 Department of Chemical Engineering, University of New Brunswick, Fredericton, NB E3B 5A3, Canada; hxiao@unb.ca

**Keywords:** nanocellulose, polyacrylic acid, polypyrrole, hydrogel, self-healing and conductive

## Abstract

Hydrogel-based strain sensors inspired by nature have attracted tremendous attention for their promising applications in advanced wearable electronics. Nevertheless, achieving a skin-like stretchable conductive hydrogel with synergistic characteristics, such as ideal stretchability, excellent sensing performance and high self-healing efficiency, remains challenging. Herein, a highly stretchable, self-healing and electro-conductive hydrogel with a hierarchically triple-network structure was developed through a facile two-step preparation process. Firstly, 2, 2, 6, 6-tetrametylpiperidine-1-oxyl (TEMPO)-oxidized cellulose nanofibrils were homogeneously dispersed into polyacrylic acid hydrogel, with the presence of ferric ions as an ionic crosslinker to synthesize TEMPO-oxidized cellulose nanofibrils/polyacrylic acid hydrogel via a one-pot free radical polymerization. A polypyrrole conductive network was then incorporated into the synthetic hydrogel matrix as the third-level gel network by polymerizing pyrrole monomers. The hierarchical 3D network was mutually interlocked through hydrogen bonds, ionic coordination interactions and physical entanglements of polymer chains to achieve the target composite hydrogels with a homogeneous texture, enhanced mechanical stretchability (elongation at break of ~890%), high viscoelasticity (maximum storage modulus of ~27.1 kPa), intrinsic self-healing ability (electrical and mechanical healing efficiencies of ~99.4% and 98.3%) and ideal electro-conductibility (~3.9 S m^−1^). The strain sensor assembled by the hybrid hydrogel, with a desired gauge factor of ~7.3, exhibits a sensitive, fast and stable current response for monitoring small/large-scale human movements in real-time, demonstrating promising applications in damage-free wearable electronics.

## 1. Introduction

Recently, the concept of mimicking human skin to develop artificial tissue-like electronic devices has drawn widespread interest due to their broad potential applications in soft robotics, wearable devices, tissue engineering, bioelectronics and artificial intelligence [1,2,3]. As the essential component of skin-inspired electronics, flexible and wearable strain sensors with a sensitivity similar to human skin tactile sensations can transduce mechanical deformations into electrical signals and generate repeatable electrical responses upon external forces, which has inspired remarkable efforts in fabricating innovative soft materials with special functional features [4,5]. To construct high-performance sensing materials, the integration of superior stretchability and self-healability is considered vital for the long-term practical application of personalized electronics [6,7]. On the one hand, the sensing materials need to be flexible and stretchable (elongation greater than 180%) to imitate the elastic and soft characteristics of human skin [8]. One the other hand, integrating self-healing ability into sensing materials can avoid damage to device performance during repeated deformations and considerably improve the durability of the electronics [9]. However, developing electro-conductive strain sensing materials with combined high stretchability and intrinsic self-healing capability still remains challenging [7,10,11,12].

As a novel type of smart hydrogel, conducting polymer hydrogels (CPHs) that integrate the advantages of hydrogels and conducting polymers are excellent candidates for strain sensing materials because of their combined electrical conductivity and mechanical compliance [13]. The inherent 3D network structure and stable conductive pathways of CPHs can achieve an electroconductive yet mechanically strong framework, which can theoretically support the diffusion and transport of small molecules, ions, charges and electrons [11,12]. Although some CPHs have previously been prepared using conducting polymers such as poly (3, 4-ethylenedioxythiophene)- poly (styrenesulfonate)(PEDOT-PSS), polythiophene (PTh), polypyrrole (PPy) and polyaniline (PANI), their stretchability is severely restricted by the intrinsic brittleness of conjugated chains of conductive polymers [14]. To further endow CPHs with self-healing capability, reversible non-covalent interactions or dynamic molecular bonds are generally incorporated into the gel network of CPHs to restore the original mechanical properties, including host-guest recognition, hydrogen bonding, hydrophobic interaction and metal coordination bonds. Among them, metal-ligand coordination interaction is considered the more appealing approach [6,15].

Polyacrylic acid (PAA) with abundant carboxyl groups can form self-healing hydrogels by virtue of metal-ligand coordination interaction and has received enormous attention. Using intrinsically conductive polymers (e.g., PPy) for fabricating self-healing CPHs is an appropriate approach. The mechanical properties of PPy-integrated PAA hydrogels are compatible with human skin, making them ideally suitable for synthesizing strain sensing materials that are plastically deformable to curvy, stretchy and dynamic surfaces [16]. However, the presence of rigid PPy will severely degrade the mechanically stretchability and flexibility of the CPHs, hindering their potential applications in soft sensors [13]. Therefore, it remains a great challenge to engineer conductive PPy into a mechanically robust, highly stretchable and intrinsically self-healable PAA-based CPH using a reasonable network design.

To resolve the abovementioned problem, incorporating various nanofillers (e.g., carbon nanomaterials and nanoclay) into the gel network is a promising approach to enhance the mechanical strength, viscoelasticity, stretchability and electroconductivity of CPHs. Among them, TEMPO-oxidized cellulose nanofibers (TOCNFs), a type of bio-based renewable natural resource, are drawing increasing attention as green reinforcing nanofillers due to their sustainability, biodegradability, modifiability, biocompatibility, high aspect ratio, excellent mechanical properties and stable dispersion in water. The introduction of C_6_ carboxylate groups and hydroxyl groups makes TOCNFs ideally suitable for mixing with PAA, especially with the presence of supramolecular metal-ligand complexation. TOCNFs inherently assemble into a hierarchical structure to achieve their interactions with the polymer network, which can further enhance the mechanical toughness and self-healing properties of the CPHs [5]. More importantly, it is encouraging to expand the use of value-added TOCNFs as an alternative building block of sensing materials for energy, science and technology, from the viewpoint of exploiting renewable and sustainable material [17].

In this study, a new type of highly stretchable and intrinsically self-healable CPH (TOCNF/PAA-PPy) with a hierarchically triple-network structure was developed via a facile two-step preparation process. TOCNFs were firstly dispersed into PAA hydrogel with the presence of ferric ions (Fe^3+^) as an ionic crosslinker to synthesize a TOCNF/PAA hydrogel matrix via a one-pot free radical polymerization. A PPy conductive network was then incorporated into the TOCNF/PAA matrix by in situ oxidative polymerization of pyrrole (Py) monomers. By taking advantage of the synergistic effect of TOCNFs, PAA and PPy, the composite hydrogels showed enhanced mechanical strength, viscoelasticity, stretchability, electroconductivity and self-healability, endowing the hydrogel-based strain sensors with promising applications in wearable electronics.

## 2. Materials and Methods

### 2.1. Materials

A commercially available bleached wood pulp (Nippon Paper Industries Co., Ltd., Tokyo, Japan) was dried overnight at 55 °C before use. 2,2,6,6-Tetramethylpiperidine-1-oxyl (TEMPO), sodium bromide (NaBr), sodium hypochlorite (NaClO), sodium hydroxide (NaOH), acrylic acid (AA), N,N’-methylenebisacrylamide (MBA), iron chloride hexahydrate (FeCl_3_·6H_2_O), pyrrole monomer (Py), ammonium persulfate (APS) and phytic acid (PA) were purchased from Shanghai Aladdin Bio-Chem Technology Co. (Shanghai, China). All reagents and solvents were analytical grade without purification.

### 2.2. Preparation of TOCNFs

TOCNFs were isolated from bleached wood pulp in the TEMPO/NaBr/NaClO system according to a previously reported method. Initially, 2.0 g of the bleached wood pulp was added into the oxidation agent, consisting of 0.033 g TEMPO, 0.33 g NaBr and 400 mL water, with continuous stirring. The oxidation was started by dropwise addition of NaClO solution (15 mmol g^−1^ of cellulose weight) and proceeded under continuous stirring, wherein the primary alcohol groups in the C_6_ position of cellulose were position-selective and regularly oxidized to carboxylic acid groups [18]. Subsequently, the pH of the reaction system was kept at 10 with 0.5 M NaOH solution. After approximately 6 hours, the oxidation reaction was quenched with methanol and the pH was adjusted to neutral by titration of 0.5 M HCl. After repeatedly washing with water by filtration, the obtained cellulose slurry was dispersed in water and then ultrasonicated at a power of 300 W for 15 min to achieve a homogeneous TOCNF colloidal aqueous suspension with a solid content of ~0.8 wt%.

### 2.3. Synthesis of TOCNF/PAA-PPy Hybrid Hydrogels

The TOCNF/PAA-PPy hybrid hydrogels were prepared via a facile two-step synthesis process (Table 1). In the first step, the TOCNF/PAA gel matrix was synthesized through free radical polymerization of AA using MBA as a chemical cross-linker, APS as an initiator, Fe^3+^ as an ionic cross-linker and TOCNFs as a reinforcing phrase. Typically, AA (6.0 g), FeCl_3_·6H_2_O (0.2 g) and MBA (0.03 g) were dissolved in 24 mL of de-ionized water with stirring, until a homogeneous mixed solution was formed. After dissolution, 15 g of TOCNFs aqueous suspension (0.8 wt%) was added into the mixed solution, followed by an ultrasonic treatment to form the polymeric precursor solution. The precursor solution was then treated with bubbling N_2_ gas to form the de-oxygenated precursor solution. The polymerization reaction was initiated by introducing APS (12 mg) and further proceeded for 36 h at 40 °C under an N_2_ environment. After gelation, the purified TOCNF/PAA hydrogel matrixes were firstly dried and then immersed into an aqueous solution of Py monomers at various concentrations (0, 0.2, 0.4, 0.6 and 0.8 mol L^−1^) for 12 h to absorb the monomers. In the second step, Py monomers were oxidatively polymerized in situ inside the TOCNF/PAA hydrogel matrix using APS as an oxidant and PA as both a cross-linker and dopant [19]. Subsequently, TOCNF/PAA hydrogel matrixes containing different amounts of Py monomers were immersed into a mixed solution of PA (0.12 mol L^−1^) and APS (1.0 mol L^−1^) to initiate in situ polymerization of Py monomers in the gel networks. After 12 h polymerization at 0 °C, the obtained TOCNF/PAA-PPy hydrogels were washed and purified with water to remove unreacted residues. Herein, the composite hydrogels with different feeding concentrations of Py monomers were designated as TOCNF/PAA-PPy-X, where X represents the concentration of Py solution during the in-situ penetration process. Additionally, the pure PAA and TOCNF/PAA composite hydrogels were synthesized through a similar synthesis process as reference samples. 

### 2.4. Characterization

Fourier transform infrared (FTIR) analyses of the hydrogel samples were performed using a Nicolet iS10 spectrometer (Thermo Scientific, Waltham, MA, USA) in the range 500~4000 cm^−1^ with a resolution of 4 cm^−1^. The densities (*ρ*, g cm^−3^) of the hydrogels were calculated from their weight and dimensions. All samples (initial weight = *W*_i_) were dried at 60 °C to a steady weight (*W*_d_). Their water content values (Wc) were obtained by the equation (Wc = (Wi – Wd)/Wi × 100%). The dynamic rheological characteristics were measured by a rheometer (HAAKE 600, Thermo Fisher Science Inc., Waltham, MA, USA) with a plate-plate geometry (25 mm in diameter) to analyze the elastic and viscous response of the hydrogels. Before measurements were taken, a dynamic strain sweep was performed from 0.1 to 100% strain to determine the Linear viscoelastic regime (LVR). A strain (γ) of 1.0% was chosen in the following rheological measurements. The rheological parameters (log mode), involving the storage modulus (*G′*) and loss modulus (*G″*), as functions of angular frequency (*ω*), were measured at *ω* = 0.01~100 rad s^−1^ at room temperature. The complex modulus (G*) was calculated by the equation (G* = (*G′*^2^ + *G″*^2^)1/2). The mechanical properties were evaluated on a universal mechanical testing machine (TY-8000B, Tianyuan Co. LTD, Yangzhou, China) at room temperature. For the tensile stress (*σ_t_*)-strain (*ε_t_*) measurements, cylindrical hydrogel samples (40 mm in length and 4 mm in diameter) were stretched at a crosshead speed of 50 mm min^-1^. Compression stress (*σ*_c_)-strain (*ε*_c_) tests were carried out on the cylindrical hydrogels with a diameter of 40 mm and a thickness of 10 mm at a crosshead speed of 20 mm/min. All the mechanical measurements were repeated six times. The electro-conductibility was measured on an electrochemical workstation (CHI 760E, CH Instruments Ins., Shanghai, China). The cylindrical hydrogel samples (40 mm in length and 4 mm in diameter) were sandwiched between two platinum plate electrodes and the resistance (*R*) derived from the linear sweep voltammetry was calculated by the equation (*R* = *U*/*I*), where *R*, *I* and *U* were the hydrogel resistance (Ω), current (A) and open circuit potential (V), respectively. The conductivities were calculated according to the equation (*σ* = *L*/(*R* × *S*)), where *σ*, *L*, *R* and *S* were the electrical conductivity (S·m^−1^), the hydrogel length (cm), resistance (Ω) and cross-sectional area of the sample (cm^2^), respectively.

Each freshly prepared cylindrical hydrogel sample was cut into halves and then the two separate parts were put together and contacted for different periods of time (2, 4 and 6 h) at room temperature in air. After a certain time for the self-healing process, the self-healed samples were subjected to uniaxial tensile measurement, leading to the maximum stress values (*σ*_t_’). The mechanical self-healing efficiency (*f*_1_) values based on the maximum stress values of self-healed (*σ*_t_’) and original (*σ*_t_) hydrogel samples were expressed by the equation (*f*_1_ = *σ*_t_′/*σ*_t_ × 100%). The electrical self-healing efficiency (*f*_2_) values based on the electrical conductivities of self-healed (*σ*’) and original (*σ*) hydrogel samples were expressed by the equation (*f*_2_ = *σ*’/*σ* × 100%).

The evaluation of the strain sensing performance of the composite hydrogels was achieved by a homemade stretching device and an electrochemical workstation. The cylindrical samples (40 mm in length and 4 mm in diameter) were stretched to up to 800% strain and the resistance values derived from the linear sweep voltammetry were recorded continuously. The gauge factor (*GF*), representing the sensitivity of a strain sensor, was calculated by the equation (*GF* = Δ*R*/*εR*_0_), where *R_0_* was the original resistance without strain, Δ*R* was the resistance change under different strains and *ε* was the applied strain. The hydrogel-based strain sensor was connected to an electrochemical workstation through two pieces of nickel foam. To monitor human motion, the assembled sensors were attached to human finger, fist, wrist and face and the output I-t curves were recorded with cyclic movements under various amplitudes.

## 3. Results and Discussion

### 3.1. Design of TOCNF/PAA-PPy Hybrid Hydrogels 

**Figure 1:**
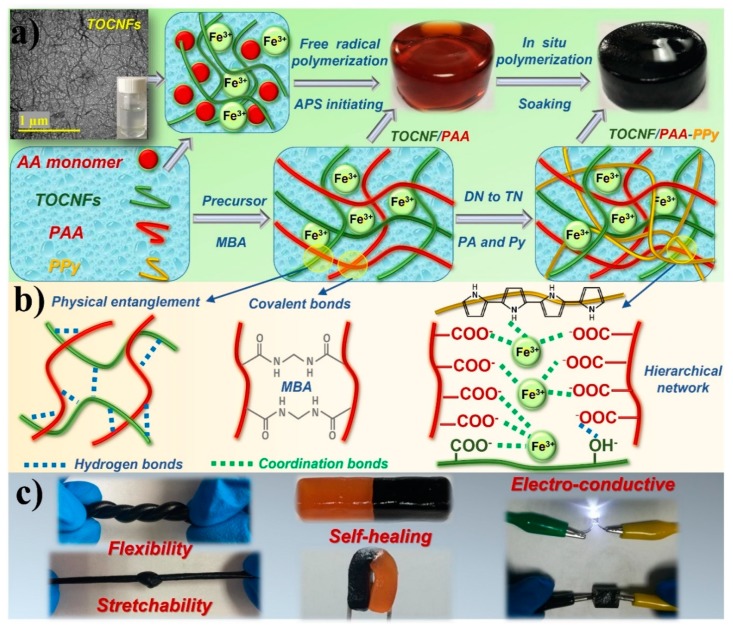
Fabrication strategy of TOCNF/PAA-PPy composite gels. (**a**) Fabrication process of the composite hydrogels. (**b**) Formation mechanism and chemical and physical interactions within the triple-network of the composite hydrogels. (**c**) Demonstration of mechanical toughness, self-healing behavior and electric-conductivity of the composite hydrogels.

The facile combined two-step preparation process of self-healing and electro-conductive TOCNF/PAA-PPy composite hydrogels with a hierarchically triple-network structure is schematically shown in Figure 1a. After the TEMPO-mediated oxidation treatment, significant amounts of C_6_ hydroxyl groups on native cellulose microfibrils were selectively oxidized to C_6_ carboxylate groups without any sacrifice of their original crystallinity [18]. Due to the strong electrostatic repulsive forces between the negatively charged carboxyl groups, as-prepared fibrous TOCNFs (3~4 nm in diameter, 1~3 μm in length, aspect ratio > 100) could be homogeneously dispersed in water to form a long-term stable colloidal suspension without any aggregation. Firstly, the double-network TOCNF/PAA hydrogel matrix was prepared via one-pot in situ free radical polymerization of AA monomers using APS as an initiator, MBA as a chemical cross-linker and Fe^3+^ as an ionic cross-linker in the presence of TOCNFs as a reinforcing phase. The chemically crosslinked PAA chains via the covalent bonds with MBA segments created a mechanically tough backbone and a first-level permanent network within the gel matrix, thus sustaining the dimensional stability of the TOCNF/PAA hydrogel [8,20]. The second-level gel network was constructed by introducing well-dispersed TOCNFs, further enhancing the mechanical strength and structural stability of the hydrogel matrix. Due to their flexibility and toughness, TOCNFs can readily deform and reform into a spatial matching configuration, enabling the chain entanglement of TOCNFs and PAA molecules. The polar hydroxyl and carboxyl groups of TOCNFs provided the interfacial compatibility and induced the formation of the physical cross-linking through the hydrogen bonding and electrostatic interaction between TOCNFs and PAA chains. Additionally, the presence of -COOH and -OH groups in the PAA and TOCNF structure built up plenty of inter- and intramolecular hydrogen bonds, thus stabilizing the double network of the TOCNF/PAA hydrogel [21]. The free trivalent Fe^3+^ cations along the TOCNFs and PAA chains created ionic cross-linking points to form dual coordination bonds between Fe^3+^ and carboxylic groups of TOCNFs and/or PAA. The construction of ionic coordination via supramolecular metal-ligand complexation led to the translucent brown color of the intermediate TOCNF/PAA hydrogel. Apart from hydrogen-bonding interaction between TOCNFs and PAA chains, the integration of Fe^3+^ caused the screening effect of inter-fiber electrostatic repulsion, making these mobile ions a physical cross-linker for chain association among TOCNFs networks. Therefore, the synergistic coordinating complexation between Fe^3+^ and TOCNFs/PAA polymer chains via metal-ligand bonds achieved the dynamic and reversible cross-linking of the hydrogel network [22]. These restorable bonds of physical cross-linking and the mobility of Fe^3+^ endowed the hydrogels with an autonomous and intrinsic self-healing capability. Afterwards, a PPy conductive network was incorporated into the TOCNF/PAA gel matrix as the third-level gel network by in situ polymerization of Py monomers, using APS as the oxidant and PA as the dopant. During the soaking and swelling process, Py gradually penetrated and diffused into the TOCNF/PAA gel matrix. The ionic interaction of Fe^3+^ ions with -COOH groups of PAA and N-H groups of Py monomers, as well as the hydrogen bonding between Py rings and TOCNFs, enabled the TOCNF/PAA hydrogel framework to serve as a favorable template for the in situ polymerization of PPy [13,21]. After being initiated by APS, the conjugated PPy chains grew gradually to form an interconnected conductive network on the basis of the TOCNF/PAA skeleton. By interacting with PPy chains, PA was partially deprotonated and interacted with PPy chains by ionic interactions and hydrogen bonds, further facilitating the development of a PPy network inside the hydrogel. The hierarchical 3D network was interlocked with each other through hydrogen bonds, ionic coordination interactions and physical entanglements of polymer chains to achieve the target TOCNF/PAA-PPy composite hydrogels with a homogeneous dark color and fine texture [23]. As expected, the obtained TOCNF/PAA-PPy hybrid hydrogels displayed a low density (~1.2 g cm^−3^), desired electro-conductivity (~3.9 S m^−1^) and high water content (~82%). Because of their texture homogeneity, mechanical toughness and elasticity, these hydrogels could be readily knotted and stretched even with a knot. The self-healed hydrogels could be randomly bent and folded without any damage on the contacting interface, demonstrating their excellent self-healing performance.

### 3.2. Morphology and Chemical Structure of Hydrogels

**Figure 2:**
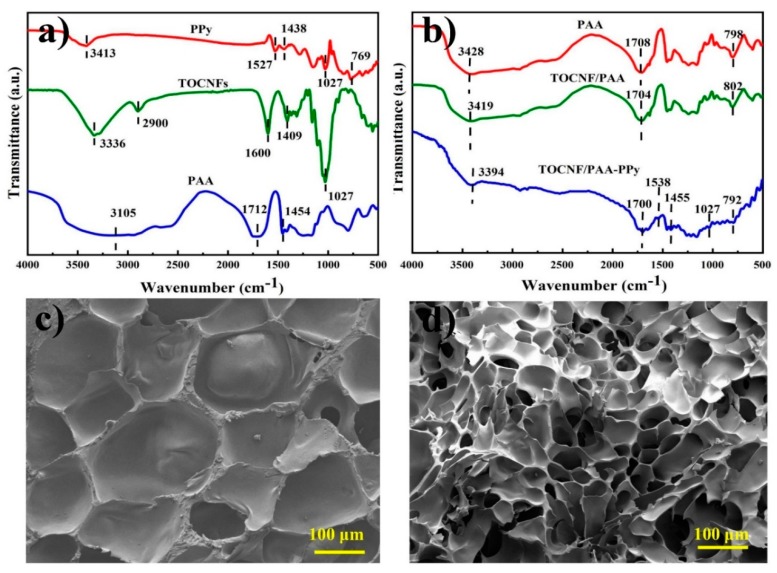
Fourier transform infrared (FTIR) spectra of pure PAA powder, PPy, TOCNFs (**a**) and PAA hydrogel, TOCNF/PAA hydrogel, TOCNF/PAA-PPy hydrogel (**b**). Scanning electron microscope (SEM) images of TOCNF/PAA hydrogel (**c**) and TOCNF/PAA-PPy composite hydrogel (**d**).

FTIR analysis was applied to characterize the chemical bonds within the hydrogels. In Figure 2a, pure PPy showed some characteristic peaks at 3413, 1527, 1438 and 1027 cm^−1^, which corresponded to the N-H stretching, C=C ring stretching band, C-N stretching vibration and =C-H vibration on the plane, respectively [24]. For TOCNFs, the broad band centered near 3336 cm^−1^ was assigned to the O-H stretching vibration and the absorption bands at 2900, 1409 and 1027 cm^−1^ were due to the C-H stretching, CH_2_ in-plane bending and CH bending vibration, respectively [25]. In the case of pure PAA, characteristic peaks at 3428, 1708 and 1454 cm^−1^ were clearly observed due to the strong and broad absorption of O-H symmetric stretching, C=O stretching and CH_2_ symmetric shearing vibrations, respectively [22,26]. Notably, the presence of carboxyl groups in the C_6_ position of TOCNFs formed during TEMPO oxidation was confirmed by the appearance of the characteristic absorption at 1600 cm^−1^. As shown in Figure 2b, the shift from 3428 to 3419 cm^−1^ indicated hydrogen bonding between the TOCNFs and PAA chains. The characteristic peak of TOCNFs due to carboxylate groups (1600 cm^−1^) was completely altered to carboxyl groups (1704 cm^−1^) by the formation of TOCNF/PAA hybrid gels [27]. This alteration might occur due to the transfer of the acidic proton of the carboxylic acid groups on PAA chains to the sodium carboxylate groups (TOCNF-COONa), which were formed by complete oxidation of the C_6_ primary hydroxyls in the TEMPO/NaBr/NaClO system [28,29]. Besides, some distinctive absorptions from 2900 cm^−1^ to 1027 cm^−1^ associated with TOCNFs disappeared in both composite hydrogels, which might be the result of the relatively low content of TOCNFs [30]. After the incorporation of PPy into the TOCNF/PAA gel matrix, the typical absorption peaks of PPy were clearly identified for TOCNF/PAA-PPy hydrogel, confirming successful in situ polymerization of Py monomers within the gel matrix. The shift of the N-H absorption peak from 3413 cm^−1^ for pure PPy to 3394 cm^−1^ for doped PPy indicated the influence of the PA doping process on nitrogen atoms [31]. For the TOCNF/PAA-PPy hydrogels, the featured peaks at 1527 (C=C stretching) and 1438 cm^−1^ (C-N stretching) of PPy red-shifted to 1538 and 1455 cm^−1^, respectively, which might be due to the hydrogen bonding between the amine groups of PPy rings and the hydrophilic groups of TOCNFs [32]. Particularly, the center peaks at 1055 cm^−1^ and 802cm^−1^ of the composite hydrogels shifted slightly towards a lower wavenumber, suggesting the interactions between PPy and PAA [33].

As shown in the scanning electron microscopy (SEM) images of Figure 2c,d, TOCNF/PAA and TOCNF/PAA-PPy composite hydrogels both presented a typical porous structure. The formation of a well-organized network without obvious agglomeration indicated a homogeneous distribution of TOCNFs in the composite hydrogels. After the introduction of PPy into the hydrogel network, the pore size was reduced due to the formation of the PPy network, which acted as a nucleating agent during the freezing process in the hydrogels [8]. Thanks to the ideal architecture, the internal stress could be effectively transferred from the PAA skeleton to the TOCNFs and PPy network under external force and the synergic reinforcing effect of the hierarchically triple-network structure provided supplemental energy dissipation ability, thus enhancing the mechanical strength of the composite hydrogels [34].

### 3.3. Dynamic Viscoelasticity of TOCNF/PAA-PPy Hybrid Hydrogels

**Figure 3:**
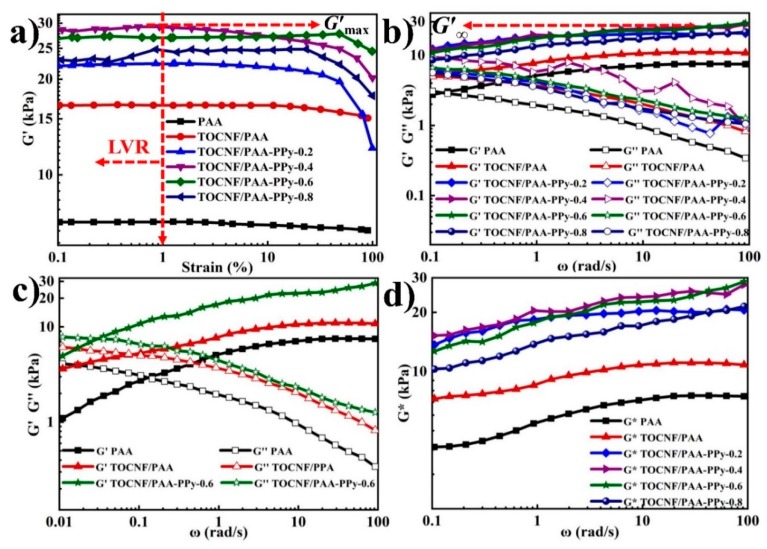
Dynamic viscoelastic behavior of various hydrogels at 25 °C. (**a**) *G’* as a function of applied strain from 0.1 to 100% at 1.0 Hz. (**b**) Oscillatory frequency sweeps from 0.1 to 100 rad/s; (**c**) Oscillatory frequency sweeps from *ω* = 0.01 to 100 rad/s; (**d**) frequency dependence of G* for hydrogels.

As shown in Figure 3a, the storage modulus (G’) as a function of strain (γ) exhibited the typical viscoelastic and solid-like nature of these hydrogels. Small amplitude oscillatory shear tests were used to demonstrate moduli basically independent of the amplitude of strain within LVR. The critical strain (c_u_) values were defined to represent the degree of deviation from the linear viscoelastic region. All the γ_c_ values ranged from 11.5% to 33.7%. The G’ values were constant at γ < 1.0% and thus a strain of 1.0% was selected for the subsequent measurements. As expected, the G’_max_ dramatically increased from 7.2 to 16.6 kPa after the incorporation of TOCNFs (Table 2), demonstrating a significant reinforcing effect of TOCNFs on the viscoelasticity of PAA hydrogels. The enhancement mechanism was mainly attributed to the strong interaction between PAA chains and TOCNFs through hydrogen bonding and chain entangling, which enabled the creation of the second-level TOCNFs network on the basis of the first PAA gel network. This phenomenon could be explained by the uniformly dispersed features and high aspect ratio of TOCNFs, which represented a large interface area per unit volume and extremely low inter-filler distance, respectively [35]. After introducing the third-level PPy gel network, the enhanced G’_max_ (~27.1 kPa) of TOCNF/PAA-PPy-0.6 was nearly 1.7 and 3.8-fold greater than those of TOCNF/PAA (~16.6 kPa) and PAA (~7.2 kPa), respectively. The hydrogen bonding interactions between PPy molecules and TOCNFs, as well as the ionic coordinating bonds between PPy molecules and PAA chains, realized the formation of the third-level PPy network on the basis of the TOCNF/PAA gel skeleton, leading to the integration of the triple-network composite hydrogels and the enhancement of their rheological viscoelasticity. As the concentration of Py increased from 0.2 to 0.8 mol L^−1^, the G’ values firstly displayed a monotonic increase, implying a constant rise in crosslinking density. However, the dynamic balance of the interlocked hierarchical network structure was destroyed due to the aggregation of excess PPy chains, leading to a decrease in G’ values. Figure 3b displays the G’ (elasticity) and G’’ (viscosity) versus ω within LVR. Overall, both G’ and G’’ values of all samples presented a dependency on ω with a comparable trend. Initially, G’ increased to reach to the high frequency plateau of G’ (G’_∞_), wherein the polymer chains were fully entangled, while G’’ reached up to its peak (G’’_max_) and then decreased gradually at a high frequency range. For all the composite gels, the G’ values were consistently larger than the counterpart G’’ at ω = 0.1~100 rad/s, demonstrating the formation of a permanent, elastic and stable hydrogel network accomplished via introducing TOCNFs and the PPy network [11,12]. To further investigate their gelation behavior, the optimized TOCNF/PAA-PPy-0.6 and the other two reference hydrogels were subjected to an oscillatory low-frequency sweep with ω starting from 0.01 rad/s (Figure 3c). The crossed gelation point of G′ and G″ curves appeared at ω=0.1~0.01 rad/s and was the sign of the gel transition from a quasi-liquid to quasi-solid state [36]. It was noticed that the occurrence of the gelation point for TOCNF/PPA-PPy-0.6 was in a lower ω than the other two gels, indicating that the hierarchical 3D gel network was established much earlier and more easily due to the incorporation of TOCNFs and PPy. The plots of G* (complex modulus) versus ω exhibited a distinct comparison of all the samples, where the G* values of TOCNF/PAA-PPy-0.6 and TOCNF/PAA-PPy-0.4 were higher than those of the other hydrogels. Considering the drastic modulus fluctuation of TOCNF/PAA-PPy-0.4, TOCNF/PAA-PPy-0.6 with a moderate amount of PPy showed the best and the most stable viscoelasticity among these composite hydrogels.

### 3.4. Mechanical Performance of Composite Gels

**Figure 4:**
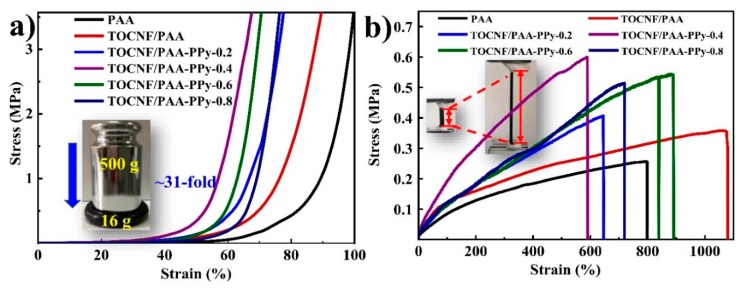
Mechanical performance of composite gels. (**a**) Compressive stress-strain and (**b**) tensile stress-strain curves of different hydrogels.

Figure 4 presents the mechanical behavior for the gels under compression and tension and the physical-mechanical performances are listed in Table 3. After the incorporation of TOCNFs, the σ_e_ value (~0.2 MPa) at ε_e_ = 60% of TOCNF/PAA composite hydrogels was two-fold larger than that of pure PAA gel (~0.1 MPa) under compression, as shown in Figure 4a, indicating that TOCNFs could significantly reinforce PAA gel through an energy dissipation mechanism based on their homogeneous distribution and intermolecular chain entanglement with PAA [37,38]. With the addition of PPy, all TOCNF/PAA-PPy composite hydrogels presented higher compressive strength than pure PAA and TOCNF/PAA hydrogels, confirming a positive impact in compressive strength through in situ polymerization of Py monomers in the TOCNF/PAA matrix. Among these composite hydrogels, TOCNF/PAA-PPy-0.4 demonstrated the highest compression strength, followed by TOCN/PPy/PAA-0.6. Further increasing the Py concentration would lead to the reduction in compression strength due to the aggregation and stress concentration caused by excessive PPy. Under tension, the pure PAA hydrogel exhibited a relatively low tensile strength of ~0.25 MPa and an elongation at break of ~795% (Figure 4b). After introducing TOCNFs into the PAA matrix, the tensile strength and elongation at break of TOCNF/PAA hybrid gels increased to ~0.36 MPa and ~1069%, respectively, due to the interlocked double-network structure of TOCNF/PAA composite hydrogels. The enhanced stretchability was ascribable to the strong interaction between TOCNFs and PAA molecular chains without sacrificing the stretchability of the composite hydrogels [39]. Similar to the compressive strength and rheological viscoelasticity, the incorporation of the PPy network could increase the tensile strength, while excessive PPy would result in a reduction in tensile strength. However, the elongations at break of TOCNF/PAA-PPy hydrogels were lower than that of TOCNF/PAA hydrogels, which might be ascribed to the intrinsic brittleness of PPy, originating from its rigid polymer backbone [40]. To balance the mechanical strength and stretchability, TOCNF/PAA-PPy-0.6 hydrogel exhibited almost the highest tensile strength of 0.55 MPa and the largest elongation at break of 889%, which makes it ideally suitable for fabricating the flexible strain sensor. Considering the high electro-conductivity of the composite gels provided by conducting PPy, the incorporation of PPy and TOCNFs could efficiently enhance the mechanical strength without sacrificing the original stretchability of PAA gels. Although PPy was selected to facilitate the electronic conductivity and promote the self-healability of the composite hydrogels, the positive synergistic effect of TOCNFs and PPy on the enhanced toughness and stable flexibility in a certain concentration range (0.2~0.6 mol L^−1^) should not be ignored [21]. Furthermore, the inherent stiffness property of PPy contributed to an inferior extension at break and an increased amount of PPy chains induced the formation of hydrogen and ionic bonding between PPy and the host TOCNF/PAA gel network. Under external force, chemical/physical cross-linkers engage in maintaining the configuration of the hydrogels, transferring load to the polymer matrix and preventing cracks from propagating, ultimately leading to a uniform hierarchical hydrogel network. However, superfluous PPy agglomeration could lead to structural disruption, stress concentration, migration resistance and resistance to the migration of PAA chains, all of which would lead to a decline of flexibility [22,41].

### 3.5. Self-Healability of TOCNF/PAA-PPy Gels

**Figure 5:**
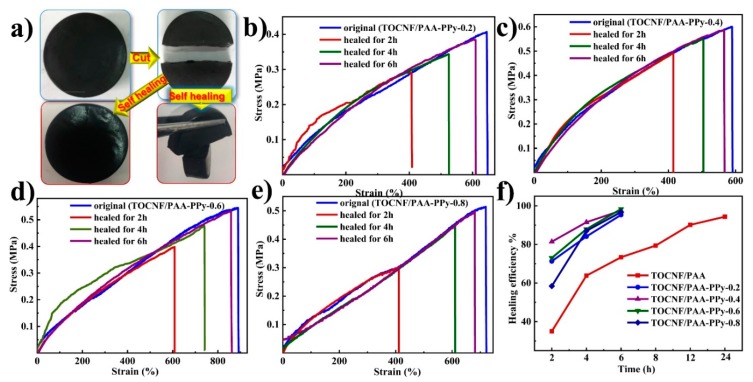
(**a**) Schematic illustration of the self-healing behavior of TOCNF/PAA-PPy gels; (**b**–**e**) Tensile stress-strain curves of the original and healed TOCNF/PAA-PPy hydrogels after different healing times; (**f**) Plots of the healing efficiency values of different composite hydrogels after different healing times.

Owing to the dynamic and reversible feature of mobile Fe^3+^ coordination sites via supramolecular metal-ligand complexation in the hierarchical hydrogel network, the prepared composite hydrogels possessed a favorable self-healing capacity at room temperature without external stimulation or reagents. As shown in Figure 5a, one piece of TOCNF/PAA-PPy hydrogel cake was equally cut into two parts with a blade. Then, the fresh cutting surfaces were in situ re-contacted in two ways. One way was complete cross-sectional re-attachment, while the other was perpendicularly re-contact. After 2 h of self-healing at room temperature, the two parts of the hydrogels gradually merged together to form a monolithic hydrogel, while the self-healed hydrogel with a perpendicular contacting surface could be readily picked up and clamped by tweezers under gravity without a fracture along the contacting interface.

To allow us to evaluate their healing efficiency, the tensile behavior of the original and self-healed gels after various healing times are plotted in Figure 5b–f. For all the composite hydrogels, the stress-strain behavior of the self-healed samples almost had the same trend and shape as the original samples, indicating the outstanding, autonomous and intrinsic self-healing performance of these composite hydrogels [42]. As the healing time increased, the maximum tensile stress and elongation at break increased accordingly for all the samples. After a 6 h self-healing process, the healing efficiency (f_1_) values (derived from maximum tensile stress) of these composite hydrogels could reach up to more than ~95%. Compared with the TOCNF/PAA hydrogels (f_1_ = 73.5% at 6 h), the TONCF/PPA-PPy-0.6 had the highest f1 value of ~98.3% at 6 h (Table 4), revealing that the incorporation of a moderate amount of PPy could significantly enhanced self-healability. This phenomenon was probably due to the coordination bonds between Fe^3+^ ions and PPy and the hydrogen bonding between PPy and TOCNFs, through which the inter-chain disruption could be spontaneously and repeatedly reconstructed. The plausible mechanism behind such a high healing efficiency of TOCNF/PPA-PPy hydrogels was proposed as follows. The intrinsic self-healing capability was attributed to the dynamically reversible crosslinked hydrogel network with a hierarchical structure. Physical crosslinking with dynamic reversible bonds dominated the self-healing behavior. The mobile trivalent Fe^3+^ ions, serving as dynamic cross-linkers, played an essential role in the self-healing mechanism [43]. One hierarchy was the dynamic nature of ionic dual coordinating interactions between Fe^3+^ ions and carboxyl (-COOH) groups on TOCNFs and PAA chains, while the other was the supramolecular metal–ligand complexation between Fe^3+^ ions and amine (-NH^+^) groups in the backbone of PPy molecular chains [21]. During the damaging-healing process, the multi-coordination could be readily restored along the disrupted polymer chains between Fe^3+^ ions and the -COOH groups of PAA and TOCNFs, as well as the -NH^+^ groups of PPy, due to the dynamic migration and reversible complexation of Fe^3+^ ions. In addition, the hydrogen bonding system and molecular chain entanglement, with dynamics and reversibility, contributed to the repetitive re-establishment of the hierarchical hydrogel network. The introduction of the second TOCNFs reinforcing network and the third PPy conductive network to the PAA hydrogel matrix could additionally form abundant high-density cross-linking sites, hence resulting in highly coiled and entangled polymer chains, which allowed a more chain sliding to occur more readily because of a decrease in interchain distances [44]. This mechanism effectively promoted the mobility of the polymer chain segments, thus enabling efficient Fe^3+^ ion diffusion and a large extension of the breakage of the re-attached interfaces [45].

### 3.6. Electro-Conductivity of TOCNF/PAA-PPy Hydrogels

**Figure 6:**
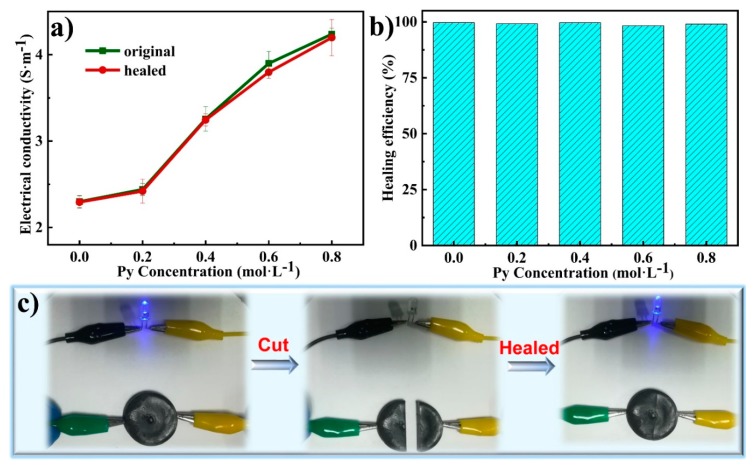
(**a**) Electro-conductivity of different composite gels before and after 6 h of self-healing. (**b**) Electrical healing efficiency of different TOCNF/PAA-PPy hydrogels. (**c**) Schematic illustration of electrical self-healability of the TOCNF/PAA-PPy hydrogels after a 6 h self-healing process in a circuit with a blue LED light.

In Figure 6a, the electroconductivity of the original gels monotonically increased from 2.4 to 4.2 S m^−1^ with the increasing Py concentration. The positive correlation between electrical conductivity and Py concentration was mainly attributed to the extraordinary electrically conductive pathway constructed by the in situ permeation and polymerization of PPy in the hydrogel network [46]. The hydrogels were endowed with high conductivity in the presence of Fe^3+^ and strong π-π stacking between the PPy conjugated backbone [46]. By virtue of the fast and efficient transportation of Fe^3+^ ions, the conductivity of TOCNF/PAA hydrogels without PPy could still reach up to 2.4 S m^−1^. The TOCNF/PAA hydrogel framework acted as a template for in situ polymerization of PPy. The Py monomers were aligned with the acrylic acid fragments, leading to regulation of growth of PPy along PAA chains and formation of a well-connected conductive path [47]. The hierarchical and high-water-retention structure encouraged the formation of abundant channel-like conduction pathways for the ion migration and electron transfer, leading to a positive effect on the integrated conductivity of the composite hydrogels. According to previous reports, CNFs could construct paths for electron and ion transportation in electrochemical composites [48]. In theory, tunnel and contact conduction were considered two principal conductive mechanisms with regard to conducting hydrogels [11]. The electrostatic repulsion caused by the carboxyl group of TOCNFs facilitates the homogeneous distribution and penetration of PPy chains, leading to an ideal 3D interconnected conducting network. The interconnected PPy conductive paths supported by the TOCNFs skeleton were suitable for accomplishing efficient tunneling current, resulting in a high electro-conductivity. Compared with the bulk sample of PPy, the 3D conductive network on the nanoscale resulted in a reduction of electrical resistance, leading to the further increase of conductivity [49]. Additionally, PA also served as a counter ion between separate PPy chains to facilitate higher elongation and ion exchanger properties, contributing to form a hierarchical porous 3D conductive nanostructure [50]. Therefore, such a hierarchically interconnected gel network not only accommodated the large PPy volumetric change during the redox processes but also provided open channels for efficient electron and ion transport.

To evaluate the self-healability of the conductive framework inside TOCNF/PAA-PPy hydrogels, the self-healing efficiency of conductivity at various Py concentrations was measured after a 6 h healing process. (Figure 6b). The electrical healing efficiency for all hydrogel samples exceeded 99%, indicating that the interconnected conducting network and electrical paths were almost completely restored after the healing process (Table 5). As demonstrated in Figure 6c, a piece of TOCNF/PAA-PPy-0.6 hydrogel was connected to a closed circuit with a blue LED. Because of the high electro-conductivity, the TOCNF/PAA-PPy hydrogel could illumine a LED bulb in a closed loop with a steady current. After the gel cake was cut into two segments, the LED was extinguished due to the open electric circuit. As expected, the LED could almost completely recover its luminance after a 6 h self-healing, demonstrating the prominent self-healing capacity and excellent electrical stability of the TOCNF/PAA-PPy hybrid hydrogels. Owing to the intrinsic self-healing capability based on dynamic reversible interactions, TOCNF/PAA-PPy hydrogels could basically maintain their original conductivity through reforming the electron transport pathways. Therefore, such an ideal electro-conductivity and self-healing property endowed the TOCNF/PAA-PPy hydrogels with great potential in the fabrication of wearable strain sensors.

### 3.7. Sensing Performance Analysis of TOCNF/PAA-PPy Based Strain Sensors

**Figure 7:**
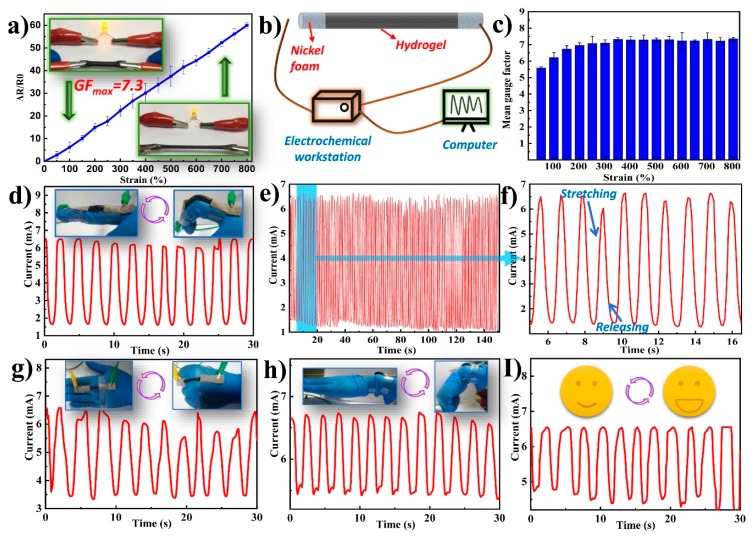
(**a**) Plot of relative variation in resistance as a function of applied strains for TOCNF/PAA-PPy-0.6 hydrogels. (**b**) Schematic diagram of the assembly of the hydrogel-based sensor. Electric current response of the strain sensor for monitoring. (**c**) Calculated mean gauge factors of the TONCF/PAA-PPy sensors under different strains between 100 and 800%. (**d**) Finger bending. (**e**) Real-time I–t curves measured by cyclic finger stretching/releasing at 5 V within 150 s for repeatability tests. (**f**) Zoomed-in version of the plot Figure 7e from 5–20 s. (**g**) Fist clenching. (**h**) Wrist bending. (**i**) Mouth opening activity at the cheek.

The integration of mechanical flexibility, stretchability, self-healing ability and conductivity makes these composite hydrogels suitable for sensing applications. To quantitatively assess the strain sensitivity of the hydrogel-based sensors, TOCNF/PAA-PPy-0.6 hydrogels were selected to assemble the strain sensors due to their high mechanical robustness and excellent electrical conductivity (Figure 7). As shown in Figure 7a, the TOCNF/PAA-PPy-0.6 hydrogel displayed a monotonic increase in the ΔR/R_0_ value against the increasing strain and the stretching process led to the darkening of the LED lighting system. This large resistance change during stretching was the foundation for high sensitivity and is highly desirable for strain sensing applications [8]. Thus, this feature essentially ensured the practical utilization of the hydrogel as an alternative sensing material, by converting mechanical stimuli into electrical signals. As shown in Figure 7a, the ΔR/R_0_ value was almost positively related to the applied strain. When the strain increased up to 800%, the corresponding ΔR/R_0_ was up to 58.6. According to the formula mentioned above (Section 2.4), the maximum value of GF was calculated to be 7.3. Based on the aforementioned GF formula, the slope of the ΔR/R_0_-strain curve represented the mean GF under the applied strain. As observed in Figure 7a,c, there were two linear regions. As the strain increased from 0% to 200%, the sensor needed to overcome a large resistance force, thus resulting in a sharp increase in resistance. This phenomenon was probably due to the combined stretching and bending deformation of the sensor, which led to structural changes such as microcracks and micropores forming in the complex hydrogels [51]. As the strain further increased from 300% to 800%, the curve appeared to show a linear regression and the GF values were stable, with only subtle fluctuations [52]. The GF value at 800% strain was comparable to those of previously reported strain sensors (Table 6) [4,8,16,23,53,54,55]. This result indicated a wide sensing range and an ideal sensitivity of the hydrogel-based sensor. More importantly, the integration of intrinsic self-healing ability and high stretchability greatly extended the long-term applications of as-prepared hydrogel-based sensors under rigorous mechanical deformations. Therefore, the sensitive, stable and fast resistance variations on the basis of mechanical deformations enabled their potential applications in detecting and monitoring human motion and physiological signals.

To further explore practical sensing applications, the TOCNF/PAA-PPy-0.6 hydrogel-based sensors—assembled with nickel foam in two ends—were linked with an electrochemical workstation and a computer to monitor the current variation caused by various human motions (Figure 7b). In Figure 7d, the sensor was attached to the joint of the index finger to detect current signals for periodical bending motions of the finger joint. With different finger bending angles, the current decreased gradually due to the increased resistance, while the current was conversely increased to the original value when the index finger completely became straight again. More importantly, the sensors exhibited a repeatable current response with periodical movements due to the mechanical elasticity and electrical stability of the hydrogels. When the volunteer kept bending their finger many times, a series of responding current wave patterns was generated accordingly, suggesting that the assembled sensor could successfully monitor continuous human movements in real-time.

To demonstrate the reliability of the TOCNF/PAA-PPy-based sensors, a cyclic bending test was conducted by laminating the sensor on the joint of the human forefinger at a bending angle of 120° up to 100 times within 150 s (Figure 7e) [56]. Figure 7f shows current changes of the sensor under repetitive stretching/releasing cycles from 5 to 20 s. It can be clearly observed that each cycle exhibited similar waveforms and the current signal could completely restore to its original value after each bending cycle, thus demonstrating the excellent cycling stability and reliability of the sensor. To further investigate their feasibility and accessibility, the strain sensors were fixed onto the back of the hand and wrist for the detection of large-scale human movements (Figure 7g,h). Apart from large-scale movement monitoring, the strain sensors were also capable of detecting tiny human motion (small-scale movements) due to their high electrical sensitivity. For example, the strain sensor mounted on the human cheek could identify and monitor the human facial expression (Figure 7i). When the volunteer opened their mouth, a slight tensile stress was generated and applied to the strain sensor due to the stretching of the muscles around the mouth. Consequently, the current signal amplitude differed from the deformation degree of the conducting networks and repetition of consistent movements led to similar current responses [2]. With regards to these regular motions, the current patterns exhibited almost consistent peaks and shapes, indicating the excellent repeatability and stability of the current response performance for the as-prepared sensors. Such a combination of high sensitivity, electrical stability and mechanical stretchability endowed the hydrogel-based strain sensors with promise for applications in wearable electronics.

## 4. Conclusions

In summary, a novel type of skin-inspired self-healing and electro-conductive TOCNF/PAA-PPy composite hydrogel with a hierarchically triple-network structure was successfully synthesized via a facile combined two-step preparation process. Firstly, the double-network TOCNF/PAA hydrogel matrix was prepared through in situ free radical polymerization of AA monomers using APS as an initiator, MBA as a chemical cross-linker and Fe^3+^ as an ionic cross-linker in the presence of TOCNFs as a reinforcing phase. Secondly, a PPy conductive network was incorporated into the TOCNF/PAA gel matrix as the third-level gel network by oxidatively polymerizing Py monomers. The ionic interaction of Fe^3+^ ions with -COOH groups of PAA and N-H groups of Py monomers, as well as the hydrogen bonding between Py rings and TOCNFs, enabled the TOCNF/PAA framework to serve as a favorable template for the integration of the PPy network. The synergistic effect of hydrogen bonding, ionic coordination interactions, polymer chain entangling and physically and chemically dual cross-linking endowed the TOCNF/PAA-PPy composite gels with a low density (~1.2 g cm^−3^), high water content (~82%), enhanced mechanical stretchability (elongation at break of ~890%), high viscoelasticity (maximum storage modulus of ~27.1 kPa), intrinsic self-healing ability (electrical and mechanical healing efficiencies of ~99.4% and 98.3% after 6 h) and ideal electro-conductibility (~3.9 S m^−1^). The hydrogel-based strain sensors, with a desired gauge factor of ~7.3, demonstrated sensitive, fast and stable electrical responses and they could consecutively monitor small/large-scale human movements in real-time, showing great potential for the design of self-healable, stretchable and skin-like sensing electronic devices.

## Figures and Tables

**Table 1:** Experimental formulas of different hydrogels.

AA (g)	H_2_O (mL)	MBA/AA (*w*/*w*)	FeCl_3_/AA (*w*/*w*)	APS (mg)	PA (g)	Py (mol L^−1^)	TOCNFs/AA (*w*/*w*)	Hydrogel Designation
6	24	0.5	0.9	12	2	0	0	PAA
6	24	0.5	0.9	12	2	0	2	TOCNF/PAA
6	24	0.5	0.9	12	2	0.2	2	TOCNF/PAA-PPy-0.2
6	24	0.5	0.9	12	2	0.4	2	TOCNF/PAA-PPy-0.4
6	24	0.5	0.9	12	2	0.6	2	TOCNF/PAA-PPy-0.6
6	24	0.5	0.9	12	2	0.8	2	TOCNF/PAA-PPy-0.8

**Table 2:** Rheological parameters derived from moduli curves.

Parameter	Gel-0.2	Gel-0.4	Gel-0.6	Gel-0.8	TOCNF/PAA	PAA
Critical strains, *γ*_c_ (%)	25.1	21.7	16.2	11.5	33.7	n.a.
*G*’_max_ (kPa)	21.1	28.9	27.1	24.4	16.6	7.2
*G*’_∞_ (kPa)	20.6	27.7	28.7	21.4	7.6	11.1

Note: (1) TOCNF/PPA-PPy is named Gel. (2) n.a. means negligible amount.

**Table 3:** Physical-mechanical properties of various gels.

Sample	*σ*_t_ (MPa)	*ε*_t_ (%)	*σ*_e_ (MPa) at *ε*_e_ = 60%	*W*c (%)	ρ (g cm^−3^)
PAA	0.25 ± 0.05	795 ± 53	0.14 ± 0.02	81.9 ± 0.5	1.1 ± 0.1
TOCNF/PAA	0.36 ± 0.03	1069 ± 26	0.23 ± 0.03	82.0 ± 0.6	1.1 ± 0.2
TOCNF/PAA-PPy-0.2	0.41 ± 0.07	644 ± 52	0.46 ± 0.04	82.0 ± 0.8	1.2 ± 0.1
TOCNF/PAA-PPy-0.4	0.60 ± 0.10	588 ± 21	1.65 ± 0.12	81.4 ± 0.7	1.2 ± 0.4
TOCNF/PAA-PPy-0.6	0.55 ± 0.05	889 ± 46	0.67 ± 0.08	82.2 ± 0.4	1.3 ± 0.2
TOCNF/PAA-PPy-0.8	0.52 ± 0.06	719 ± 33	0.24 ± 0.03	82.1 ± 0.6	1.2 ± 0.1

**Table 4:** Mechanical self-healing efficiency of various hydrogels after different healing times.

Sample	*f*_1_ After 2 h Healing	*f*_1_ After 4 h Healing	*f*_1_ After 6 h Healing
TOCNF/PAA	28.9%	64.6%	73.5%
TOCNF/PAA-PPy-0.2	71.3%	84.1%	95.3%
TOCNF/PAA-PPy-0.4	81.4%	91.5%	97.5%
TOCNF/PAA-PPy-0.6	73.0%	87.9%	98.3%
TOCNF/PAA-PPy-0.8	58.4%	87.0%	96.7%

**Table 5:** Electrical self-healing efficiencies of different hydrogels.

Sample	*σ*_R_ (S m^−1^)	*σ*’_R_ (S m^−1^)	*ƒ*_2_ (%)
PAA	2.3 ± 0.1	2.3 ± 0.1	99.5
TOCNF/PAA	2.3 ± 0.1	2.3 ± 0.2	99.5
TOCNF/PAA-PPy-0.2	2.4 ± 0.2	2.4 ± 0.1	99.1
TOCNF/PAA-PPy-0.4	3.2 ± 0.1	3.2 ± 0.2	98.7
TOCNF/PAA-PPy-0.6	3.9 ± 0.2	3.8 ± 0.1	99.4
TOCNF/PAA-PPy-0.8	4.2 ± 0.1	4.2 ± 0.3	99.0

**Table 6:** Previously reported Gauge Factor (GF) values of hydrogels strain sensors.

Material	Gauge Factor	Linearity
PAAerGO nanocomposite [8]	0.31–1.32	nonlinear
PAA-PANI [16]	0.60–1.05	two linear regions
PANI-PAA [23]	4.7–11.6	two linear regions
CNCs, PVA and PVP [4]	~0.478	nonlinear
κ-carrageenan/PAAm DN [53]	~0.63	nonlinear
PVA-PAA-MNPs [54]	~0.06	linear
Fibers-silicone [55]	~0.348	nonlinear
This work	~7.3	two linear regions
